# Metal overload synergy induces cell death in gastric cancer

**DOI:** 10.1038/s41418-025-01651-z

**Published:** 2025-12-19

**Authors:** Mitrajit Ghosh, Philipp A. Lang

**Affiliations:** Department of Molecular Medicine II, Medical Faculty, Heinrich Heine University, Düsseldorf, Germany

**Keywords:** Cancer microenvironment, Cancer models

Cancer cells often stand refractory to multiple types of therapy. In recent years, metal ion therapy in cancer to induce cell death has drawn significant interest. Manipulating metal ion homeostasis in cancer cells can disrupt protein function, induce oxidative stress, and trigger a cascade of cellular damage. Particularly, roles and mechanisms of calcium ion-mediated mitochondrial death, iron ion-mediated ferroptosis, and copper ion-mediated cuproptosis have been shown in several studies to induce tumor cell demise. This provides novel insights for targeted regulation and therapeutic intervention [1]. On the other hand, Gastric cancer (GC) is the fourth leading cause of cancer deaths worldwide and the fifth most frequently diagnosed cancer, making it a significant global health burden. Despite the advancements in targeted therapy and immunotherapy, the overall prognosis for gastric cancer remains poor, and thus, there is an unmet need for effective tailored therapy for superior clinical outcomes.

In the manuscript titled ‘MTF1 attenuates ferroptosis and cuproptosis synergistic potentiation in gastric cancer’ published in the present issue of *Cell Death and Differentiation*, the Jia group addresses the complex interplay between ferroptosis and cuproptosis and provides novel insights on molecular drivers of intrinsic mechanisms that are regulated by metal-regulatory transcription factor 1 (MTF1) in a GC setting [2].

Rapidly proliferating tumor cells have enhanced iron uptake capacity to meet the metabolic needs of a growing tumor, thus making them more susceptible to iron overload-mediated cytotoxicity [3]. Overloaded iron ions trigger ferroptosis, which plays an important role in tumor therapy. The iron ion-dependent regulated cell death or ferroptosis in tumor cells is achieved under iron overloading conditions, when Fe^2+^ overload exceeds the ferritin buffer capacity, nuclear receptor coactivator 4 (NCOA4), a specific cargo receptor of ferritin, can degrade it and release abundant Fe2+ into the cytoplasm. Free Fe^2+^ in the labile iron pool within tumor cells generates substantial hydroxyl radicals via the Fenton reaction (Fe^2+^ + H_2_O_2_ → Fe^3+^ + ·OH + OH^−^). These ·OH radicals, armed with strong oxidizing properties, specifically attack polyunsaturated fatty acid (PUFA) phospholipids abundant in the tumor cell membrane, initiating a chain reaction of lipid peroxidation [4]. When the level of lipid peroxidation exceeds the scavenging capacity of the tumor cell’s antioxidant systems, the structural integrity of the cell membrane is compromised, and membrane permeability increases, leading to ferroptosis of the tumor cell. Ferroptosis induced by iron ion overloading can increase the sensitivity of tumors to radiotherapy, chemotherapy, and immunotherapy, overcoming treatment resistance [5].

Overloaded copper ions kill tumor cells not through traditional oxidative stress, but via a cascade of “proteotoxic stress-mitochondrial dysfunction” known as cuproptosis, which specifically targets proteins associated with the mitochondrial tricarboxylic acid (TCA) cycle. Excessive Cu^+^ enters mitochondria and induces disulfide bond-dependent oligomerization of lipoylated proteins, directly blocking substrate transfer in the TCA cycle and causing disruption of cellular energy supply [6]. Ferredoxin 1 (FDX1), an upstream regulator of cuproptosis, not only mediates lipoylation modification but also reduces intracellular Cu^2+^ to Cu^+^. Excessive Cu^+^ competitively binds to Fe-S cluster proteins, leading to the dissociation of Fe-S clusters [7]. Since Fe-S clusters are pivotal cofactors for maintaining the enzymatic activity of these proteins, their disruption further inhibits mitochondrial respiratory chain function, leading to mitochondrial membrane potential depolarization and promoting the opening of mitochondrial permeability transition pores (mPTP), thus releasing apoptosis-related factors such as cytochrome C.

**Fig. 1:**
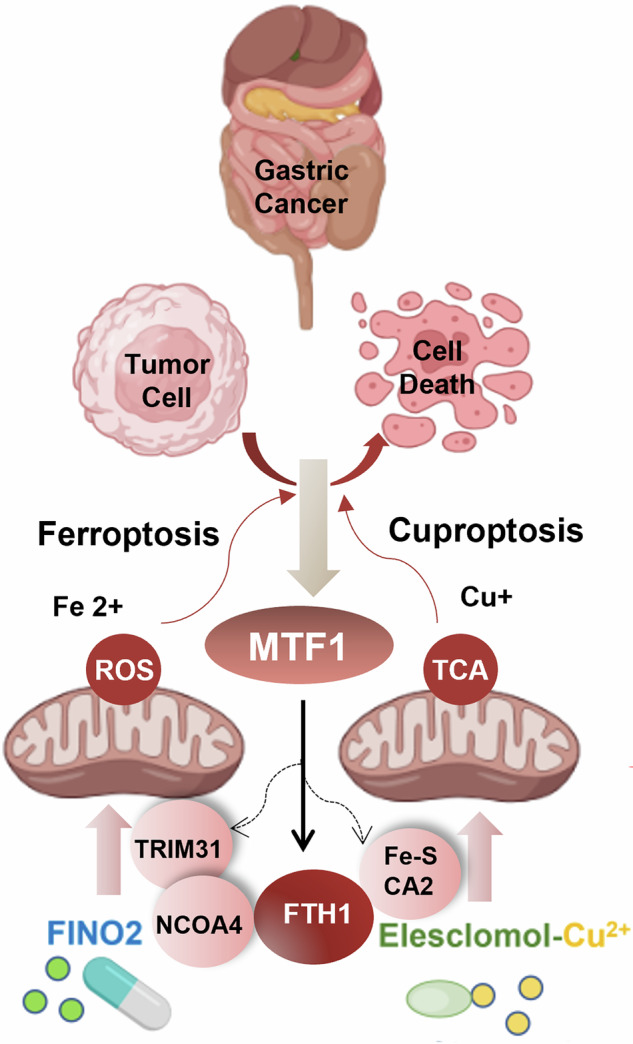
MTF1 blocks synergistic cytotoxicity potential of ferroptosis and cuproptosis in gastric cancer. FINO2 induces ferroptosis in GC by iron overloading and eventually making free Fe^2+^ available in the labile iron pool within the tumor cell that generates OH radicals, which in turn induce lipid peroxidation, ultimately leading to cell membrane disintegration and death. ES-Cu, on the other hand, induces cuproptosis in GC by blocking substrate transfer in the TCA cycle and causing disruption of cellular energy supply, and excessive Cu^+^ competitively binds to Fe-S cluster proteins, leading to the dissociation of Fe-S clusters, ultimately leading to cell death. As such, the combination treatment can enhance sensitivity to ferroptosis and cuproptosis in GC, leading to tumor cell death. MTF1 is the key gene blocking the efficacy of the FINO2/ES-Cu induced cell death. MTF1 suppresses ferroptosis by transcriptional upregulation of FTH1, which reduces Fe^2+^ levels, and MTF1 also activates TRIM31, which catalyzes ubiquitination of NCOA4, which then promotes expression of FTH1. MTF1 also activated Fe-S cluster assembly 2 (CA2)-mediated Fe-S cluster assembly and iron starvation response to inhibit cuproptosis and ferroptosis. Figure Created with BioRender.com.

In this study by the Jia group, they explored the intriguing combinatorial anti-tumor approach from ferroptosis and cuproptosis, as there is compelling evidence showing an intricate link between these two processes in GC. To determine whether ferroptosis and cuproptosis exert a synergistic effect, they combined FINO2 (ferroptosis inducer) [8] and ES-Cu (cuproptosis inducer) [9] to harness the potential anti-tumor effect for GC. Mechanistically, they identify that FINO2 and the Es-Cu combination can accelerate sensitivity to ferroptosis and cuproptosis, which could be blocked by MTF1, a transcription factor that maintains metal ion homeostasis and resists cell injury by excess metal ions [10]. Several studies have indicated that MTF1 may be an intermediate link to ferroptosis and cuproptosis [11, 12]. However, the precise role of MTF1 in GC progression and the underlying mechanisms was not known. The authors of this paper found MTF1 inhibited FINO2/ES-Cu-induced ferroptosis via upregulation of FTH1 to reduce Fe^2+^ levels. In addition to direct transcriptional upregulation of FTH1, MTF1 activated TRIM31, which consequently catalyzed ubiquitination of NCOA4 and also enhanced the expression of FTH1. On the other hand, it also activated Fe-S cluster assembly 2 (CA2)-mediated Fe-S cluster assembly and iron starvation response to inhibit cuproptosis and ferroptosis (Fig. 1). Using in vitro GC cells, in vivo GC xenograft mice models, and GC patient samples, this study elegantly dissects the mechanistic underpinnings of synergistic iron and copper overload-mediated cytotoxicity of tumor cells. This approach can eventually open new doors for innovative combinatorial therapies to obtain maximum benefits for anti-tumoral efficacy and for potential MTF1 targeted therapy.

Several other overloaded metal ions, such as Na, Mn, Zn, and Ca, have also been used in the context of anti-tumor therapy to exert different biological effects, such as initiation of death pathways, disruption of organelle functions, recruitment and remodeling of the tumor immune microenvironment [13]. To capitalize on the efficient synergistic anti-tumor activity, multimetallic ions strategies could be harnessed through functional complementarity and mechanism superposition, leading to cascade effects from induction of cell death to immune activation for superior therapeutic efficacy. In this direction, exploration of nanoplatforms-based ion overload regulation holds enormous promise for targeted tumor therapy and drug release in response to specific environmental stimuli. This approach would enhance treatment efficacy and also prevent resistance to other treatments, and therefore, this promising field needs full exploration in the near future. However, despite the encouraging potential of overloaded metal ions in anti-tumor strategies, their clinical translation faces multiple challenges and risks that need systematic consideration. Non-specific metal ion accumulation in normal tissues is a major hindrance to consider, as it can trigger adverse side effects and the nanocarriers used for the delivery of ions, which can trigger immune responses that limit their biocompatibility [14, 15]. Optimal and controlled regulation of intracellular metal ion concentrations is critical in the complex and heterogeneous tumor microenvironment. Insufficient release of metal ions will not induce overload-mediated tumor cell death, whereas excessive release exacerbates systemic toxicity. Stimuli-responsive release systems often suffer from inconsistent responsiveness in heterogeneous TMEs, leading to uneven ion distribution within tumors that may result in incomplete tumor regression and recurrence. The complex TME, such as dense extracellular matrix and abnormal vasculature, hinders the accumulation and deep penetration of metal ion carriers in tumors, and even with ligand-modified targeted nanocarriers, the binding site barrier effect may prevent successful sufficient ion delivery to inner tumor cells, reducing therapeutic efficacy and increasing the risk of drug resistance. Therefore, to achieve maximum therapeutic benefit, further research should address multiple synergistic ion overload strategies along with improved designed nanoparticles for optimal selective delivery and reduced adverse reaction.
